# DHAFGan: A dense hybrid attention fusion generative adversarial network for infrared and visible image fusion

**DOI:** 10.1371/journal.pone.0346471

**Published:** 2026-09-11

**Authors:** Qiong Hong, Zhonghua Xu, Dongli Qin, Yuhui Zheng, Hao Zhang

**Affiliations:** 1 Business School, Jiangsu Vocational College of Electronics and Information, Huaian, China; 2 School of Transportation Engineering, Huai’an University, Huaian, China; 3 Smart Campus Service Center, Jiangsu Vocational College of Finance and Economics, Huai’an, China; 4 School of Artificial Intelligence, Nanjing University of Information Science and Technology, Nanjing, China; Sichuan University, CHINA

## Abstract

Aiming at the problems existing in the current infrared and visible light image fusion algorithms, such as insufficient perception of typical features, poor visual representation of the fusion results, and insufficient utilization of important secondary information, this paper proposes an infrared and visible light image fusion algorithm based on shallow-deep feature extraction and dual-channel hybrid attention. Firstly, a shallow-deep feature extraction module is constructed. This module utilizes shallow convolutional layers and deep multi-scale receptive field units to extract surface-level features and deep semantic information from the source images, respectively, thereby achieving multi-level multimodal feature extraction. Secondly, Dual-Channel Hybrid Attention Fusion Module (DCAFM) is constructed. Spatial attention is focused on the salient areas of the image, and channel attention is used to strengthen the feature channels, thereby enhancing the fusion ability of multimodal features. Finally, primary and secondary feature loss functions are formulated to constrain both the generator and discriminator, facilitating the extraction of latent secondary feature information from the source images. Experimental results on the DroneVehicle dataset demonstrate that the proposed algorithm achieves superior performance in both subjective visual evaluation and objective metrics. Quantitative evaluations show that our method outperforms seven state-of-the-art approaches, achieving the highest scores in standard deviation (SD = 9.3541), mutual information (MI = 2.4321), and peak signal-to-noise ratio (PSNR = 65.7852), while ranking second in average gradient (AG = 3.9854). The fused images generated by our method not only align with human visual perception characteristics but also retain rich detailed information, effectively preserving both dominant and subtle features from the source modalities.

## Introduction

With the continuous development of information perception technology, multimodal image fusion has become a key approach to improving scene understanding and perception. As an important direction in this field, infrared and visible image fusion combines the advantages of infrared images, which are sensitive to thermal targets, and visible images, which provide clear texture details, to achieve a more complete and accurate representation of complex scenes. After decades of development, this technology has formed a relatively complete fusion framework and has given rise to a variety of typical algorithms. It shows broad application prospects in areas such as intelligent perception, remote sensing monitoring, and smart cities. Fusion algorithms can be divided into traditional methods and those based on deep learning.

Traditional infrared and visible image fusion algorithms typically follow a three-stage pipeline: image feature extraction, feature fusion, and image reconstruction, which are executed progressively. Depending on the transformation methods employed, traditional approaches primarily include multi-scale decomposition, sparse representation, subspace-based methods, saliency-based techniques, and hybrid algorithms. Fusion algorithms based on multi-scale decomposition are widely used for multimodal images [1, 2]. These methods typically involve three steps: first, performing multi-scale decomposition on the source image; second, processing the decomposed features according to specific fusion rules; and finally, reconstructing the fused image through an inverse transformation. Common transformation methods include pyramid transforms, non-subsampled contourlet transforms, and edge-preserving filters, among others. For instance, Jin et al. [3] developed an improved Laplacian pyramid transform by calculating the ratio between adjacent low-pass filtered images in the Gaussian pyramid, thereby enhancing local contrast information extraction. Zuo et al. [4] decomposed the source image using a region segmentation strategy within the two-tree discrete wavelet transform, enhancing the algorithm’s stability and enabling irregular sampling. However, the inherent limitations of two-dimensional wavelet transforms make it difficult to fully capture the rich directional information present in source images. In this regard, Meng et al. [5] employed non-subsampled contourlet transform to achieve multi-directional information extraction and fusion, which better preserves texture details in the resultant image, albeit with some residual visual artifacts. To further mitigate visual artifacts, Ma et al. [6] proposed a fusion method based on Gaussian and rolling guidance filters. This approach reduces visual artifacts while enhancing the overall visual quality, producing results that align more closely with human visual perception. However, multi-scale decomposition-based fusion algorithms remain susceptible to deviations in fusion results caused by image registration errors and noise. Further optimization efforts have led to the exploration of sparse representation-based fusion algorithms. These methods have garnered significant attention from researchers worldwide due to their ability to learn dictionaries from large sets of natural images, thereby enhancing image representation. For example, Liu et al. [7] developed an adaptive sparse representation algorithm that replaces traditional fixed dictionaries with sub-dictionaries learned directly from source images. This approach not only reduces visual artifacts in the fused image but also lowers computational costs. Subspace-based fusion algorithms map high-dimensional image information into lower-dimensional subspaces. This process reduces redundant information while enhancing the generalization capability of the fusion model. Prominent techniques in this category include principal component analysis (PCA) [8–10], independent component analysis (ICA) [11,12], and non-negative matrix factorization (NMF) [13,14]. These algorithms typically employ a sliding window technique for image decomposition. Saliency-based fusion algorithms prioritize salient regions and the objects within them. It mainly includes two categories: the calculation of salient feature weights and the extraction of salient features from feature maps. For instance, Ma et al. [6] significantly enhanced contrast in the fused image by extracting target weights from source images using visual saliency maps. Zhang et al. [15]addressed the problem of imbalanced information retention by constructing saliency maps to guide feature fusion. Recognizing the respective advantages of the above-mentioned algorithms, some scholars have comprehensively improved the fusion effect by conducting algorithm fusion. For example, hybrid algorithms integrating multi-scale transformation with saliency detection [16,17] can highlight object information in salient regions while simultaneously preserving detailed texture information.

In recent years, the successful application of deep learning in computer vision and image processing has led to its widespread adoption for image fusion. Deep learning-based methods, which leverage neural networks to autonomously extract features and enhance the adaptability of fusion algorithms, have attracted significant scholarly interest. Pioneering work by Li et al. [18] introduced an autoencoder-based framework for feature extraction in image fusion. However, the reliance on L1-norm-based fusion rules limited the performance and adaptability of this approach. Zhang et al. [19] enhanced the retention of multimodal image information by incorporating dense connections and multi-scale attention mechanisms into the autoencoder. Liu et al. [20] were among the first to integrate convolutional neural networks (CNNs) into the feature fusion stage. Nevertheless, the continued dependence on hand-crafted fusion rules for feature extraction ultimately constrained the fusion performance. To address these limitations, Xu et al. [21] proposed an end-to-end fusion algorithm by introducing a consistency measurement loss, which mitigated the challenge of manual weight allocation during fusion. However, the relatively simple network architecture of this model limited its capacity for extracting deep image features. Ma et al. [22]first introduced the idea of adversarial game into the field of image fusion and proposed the FusionGAN algorithm. Xing et al. [23]developed a network architecture integrating a Variational Autoencoder (VAE) compression framework, achieving multi-channel fusion alongside a higher compression ratio. This approach not only balances the fusion results but also improves the training stability of the GAN. The image fusion algorithm based on Transformer has become an important research direction in the fields of image processing and multimodal fusion. Shi et al. [24] balanced the color distribution in fused images by designing a global-local dual adversarial loss and further eliminated color interference using a dedicated color loss function. Yao et al. [25] enhanced colors in low-light regions, addressed excessive noise through data synthesis and tailored loss functions, and significantly improved model fusion efficiency. This algorithm not only improves the quality of image fusion but also enhances the generalization performance of the algorithm.

To sum up, although traditional image fusion algorithms achieve image fusion through mathematical transformation and artificially designed rules, reducing the requirements for hardware to a certain extent, their limitations are becoming increasingly obvious due to the influence of image registration and image noise. Deep learning, leveraging its advantages in adaptive feature extraction and processing efficiency, has emerged as a prominent research focus in the image fusion field. However, several challenging issues persist in existing deep learning-based methods that require urgent attention.

1) Inherent differences in scene information captured by infrared and visible sensors pose a challenge. The prevalent use of sequential convolutional layers for feature extraction often fails to adequately capture the complementary information from both modalities. This leads to insufficient perception of objects in salient regions and difficulties in simultaneously preserving global intensity distribution from infrared images and fine texture details from visible images.
2) Fused images are often prone to visual artifacts, which arise from both the inherent limitations of multi-source sensors and the instability of algorithm training. Consequently, despite achieving promising results on quantitative metrics, the perceptual quality of the fused image for human observers can be unsatisfactory, failing to meet the requirements for high-quality fusion.
3) Existing algorithms primarily rely on loss functions to enforce consistency of multi-source features in the fused image. However, in practical scenarios, depending on the conditions, visible images may sometimes reveal more distinct target features, while infrared images might occasionally provide clearer texture details. Such important, albeit secondary, information is often overlooked during the fusion process.

To tackle the aforementioned issues, this paper proposes a novel infrared and visible image fusion algorithm named DHAFGan, which integrates Shallow-Deep Feature Extraction and a Dual-channel Hybrid Attention mechanism. Firstly, a Shallow-Deep Feature Extraction (SDFE) module is designed to enable simultaneous learning of low-level details and deep semantic information through multi-receptive field processing. Secondly, a Dual-channel Hybrid Attention Fusion Module (DCAFM) is developed. This module decomposes features along spatial and channel dimensions for hierarchical fusion, facilitating the mining of important secondary information from the source images. Furthermore, a dual-discriminator architecture, guided by primary and secondary feature loss functions, is employed to enhance the complementarity of heterogeneous image information. Finally, Comprehensive experiments verify that the proposed DHAFGan algorithm generates fused images that are visually natural, rich in detail, and effectively preserve both primary and secondary information from the source images.

The main contributions of this study are summarized as follows:

1) We design a novel Shallow-Deep Feature Extraction (SDFE) module that enhances the perception of target information in salient regions. This leads to more abundant and accurate information representation in key areas of the fused image, thereby facilitating improved performance in downstream applications like target recognition and detection.
2) The proposed method effectively addresses the suboptimal human perceptual quality often associated with traditional fusion algorithms. The generated fused images exhibit more natural and clearer visual effects with reduced artifacts, thereby meeting the demands for high-quality visual perception and broadening the application scope of fused images in vision-related tasks.
3) We propose a Dual-channel Hybrid Attention Fusion Module (DCAFM) that enables the fused image to fully utilize important secondary information from less salient areas of the source images. This includes unique textures and details from either modality that may be prominent under specific conditions. This not only enriches the informational content of the fused result but also enhances its adaptability and robustness in complex and varying scenes.
4) We introduce a dual-discriminator adversarial training framework, optimized with primary and secondary feature loss functions. This design enhances the complementarity of heterogeneous image information. This ensures that the fused image retains critical source information while achieving a better balance of contributions from different modalities. Consequently, it generates high-quality, information-complete fused images, providing a more reliable foundation for subsequent image analysis and processing tasks.

## Related work

### Overall framework of the algorithm

**Fig 1:**
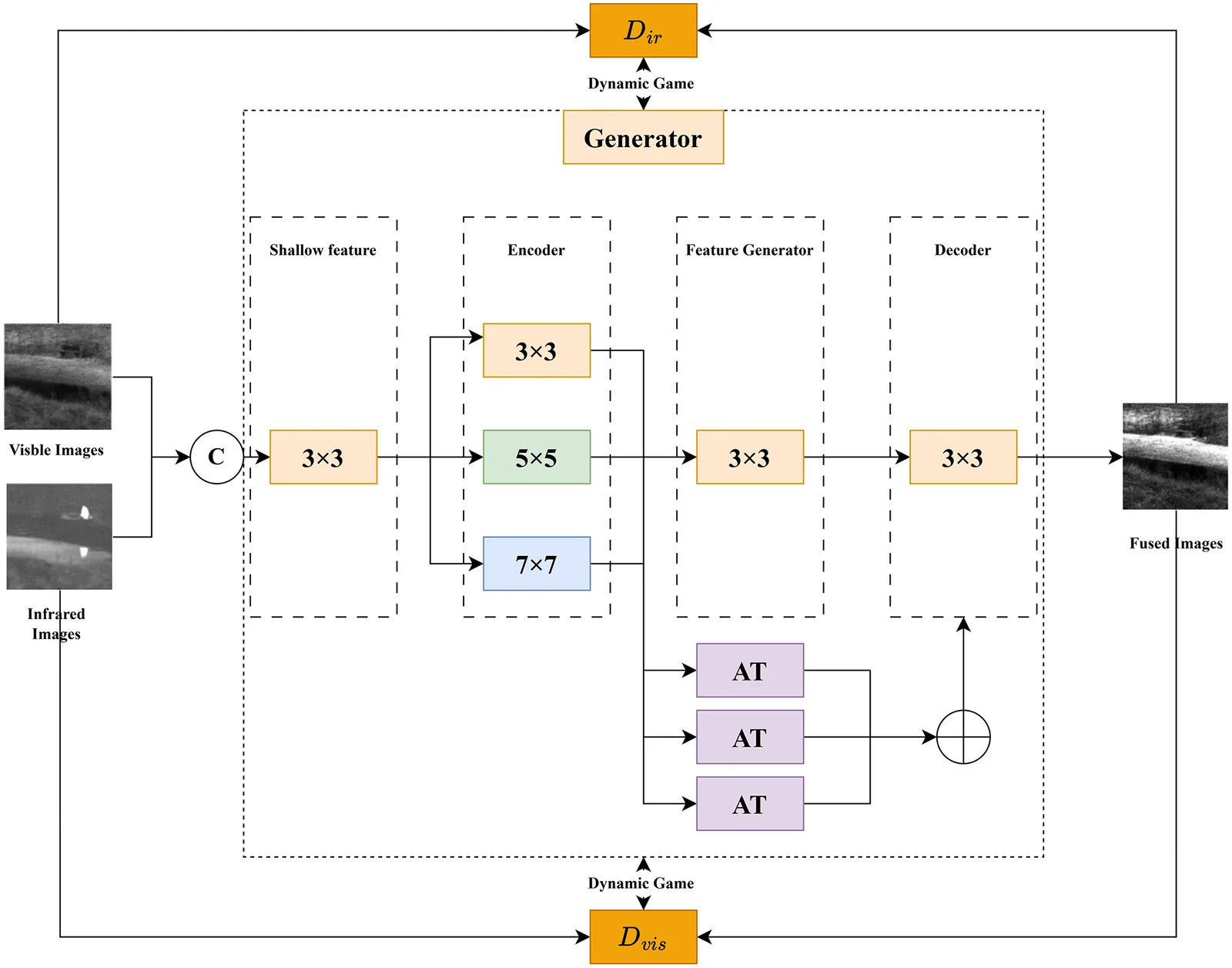
DHAFGan algorithm overall frame diagram.

As shown in Fig 1, the proposed DHAFGan framework consists of a generator and two discriminators, $D_{ir}$ and $D_{vis}$. The generator comprises a shallow-deep feature extraction module, a dual-channel hybrid attention fusion module, and a decoder, which together form an end-to-end fusion pipeline. The paired infrared and visible images are first concatenated and passed to the encoder, allowing complementary information from the two modalities to be represented jointly from the input stage. This early-fusion strategy provides the network with initial cross-modal awareness and allows the generated image to retain infrared intensity cues and visible-light gradient information even without discriminator-based constraints. The encoder subsequently extracts multi-scale deep representations through three parallel convolutional branches with kernel sizes of 3 × 3, 5 × 5, and 7 × 7. After branch-specific feature enhancement, the resulting representations are concatenated and passed to the feature fusion module for multi-scale aggregation. The decoder then reconstructs these fused representations into an image containing complementary information from both modalities.

The generator further incorporates a dual-channel hybrid attention fusion module to selectively enhance modality-specific information. Because infrared images primarily convey the intensity characteristics of salient targets, whereas visible images provide rich gradient and texture information, separate joint spatial-channel attention pathways are applied to the two modalities. The multi-scale representations extracted by the three receptive-field branches are processed in parallel to strengthen informative responses and suppress redundant information along both the spatial and channel dimensions. During decoding, a hierarchical fusion strategy integrates attention-weighted features across receptive-field scales, thereby maintaining an efficient information flow while limiting feature loss. The generator and the two discriminators are trained within an adversarial learning framework. The infrared discriminator, $D_{ir}$, imposes pixel-level intensity constraints that encourage the fused image to preserve the radiometric characteristics of the infrared source image. In contrast, the visible-light discriminator, $D_{vis}$, applies texture-fidelity constraints to preserve fine structural and textural details from the visible source image. Adversarial training approaches equilibrium when neither discriminator can reliably distinguish the generated image from its corresponding source image, indicating that the fused output satisfies the intended complementary constraints.

### Generator network architecture

**Fig 2:**
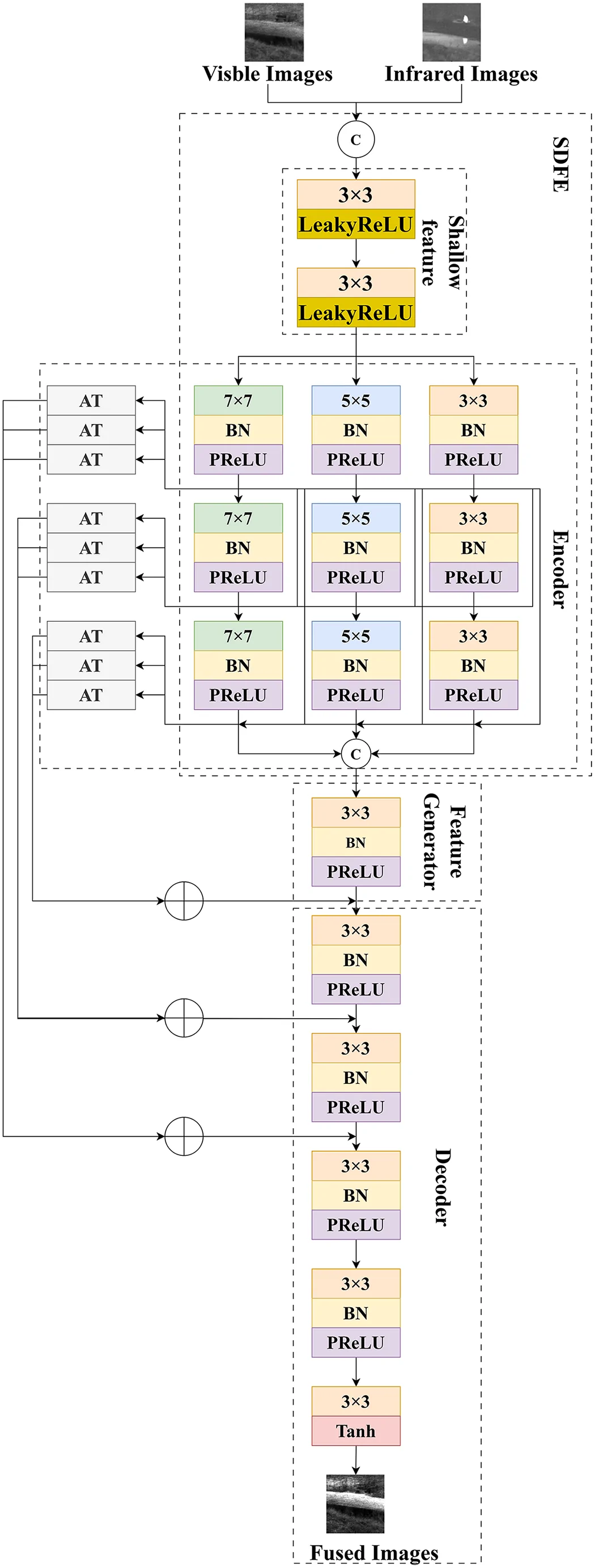
Generator network structure diagram.

As shown in Fig 2, the proposed generator comprises a shallow-deep feature extraction module, a dual-channel hybrid attention fusion module, and a decoder connected sequentially. Within the end-to-end image generation framework, this cascaded architecture performs multi-scale feature extraction, attention-guided feature fusion, and image reconstruction in sequence, thereby facilitating the effective interaction and aggregation of information from the infrared and visible modalities. The generator is designed to balance computational efficiency with the preservation of target-contrast information from the infrared source image and scene-texture details from the visible source image, ultimately producing a high-quality fused image that integrates complementary information from both modalities.

### ShAllow-Deep Feature Extraction Module (SDFE)

1) Shallow feature extraction

As shown in Fig 2, the shallow feature extraction module serves as the first processing stage of the generator and transforms the input source images into initial feature representations. The module comprises two convolutional layers with 3 × 3 kernels and a stride of 1, each followed by a LeakyReLU activation function. By applying local spatial filtering to the input images, the convolution operations capture local intensity variations and structural patterns, thereby extracting low-level visual features such as edges and textures and projecting them into a higher-dimensional feature space. This initial encoding process enhances the representational capacity of the shallow features and provides a foundational feature representation for the subsequent multi-receptive-field deep feature extraction and feature fusion stages. The shallow feature mapping process is defined as follows:


$$\begin{array}{c} \left\{F_{SF}^{\text{1}},F_{SF}^{\text{2}}\right\}=H_{SE}\left(I_{\text{1}}\right),H_{SE}\left(I_{\text{2}}\right) \end{array}$$ (1)

Where $F_{\text{SF}}^{\text{1}}$ and $F_{\text{SF}}^{\text{2}}$ denote the shallow feature representations extracted from the visible image $I_{\text{1}}$ and the infrared image $I_{\text{2}}$, respectively. $H_{\text{SE}}(\cdot )$ denotes the shallow feature extraction operation composed of a 3 × 3 convolution and a LeakyReLU activation function. After shallow feature extraction, the resulting feature maps $F_{\text{SF}}^{\text{1}}$ and $F_{\text{SF}}^{\text{2}}$ are passed to the multi-receptive-field deep feature extraction module for further processing. This module employs a cascaded convolutional structure to progressively aggregate information within local receptive fields. Specifically, each convolutional layer applies a learnable weighted summation to feature responses within local neighborhoods using sliding kernels, followed by a nonlinear activation function for feature transformation. By stacking these operations, the network progressively expands its effective receptive field and constructs hierarchical deep feature representations while preserving their spatial structure, thereby improving the model’s sensitivity to multi-scale feature patterns.

2) Multi-receptive field deep feature extraction encoder

Optimizing the performance of an image fusion algorithm requires an efficient encoder architecture that can accurately extract essential features from the source images and construct comprehensive feature representations. Previous studies have shown that the feature extraction capability of the encoder directly determines the upper bound of fused-image quality [26]. Existing deep learning methods commonly improve multi-scale feature extraction by increasing network depth and channel capacity. Although these approaches can expand the receptive field and capture deep semantic information, increasing the network scale results in exponential growth in the number of parameters and may cause training instability, such as vanishing gradients. Moreover, information attenuation during cross-layer feature propagation may lead to degradation of the fusion results. Therefore, encoder design must achieve an appropriate balance between representational capacity and computational efficiency.

To improve the completeness of feature extraction from the source images and expand the receptive-field coverage, we construct a multi-scale feature extraction module containing 3 × 3, 5 × 5, and 7 × 7 convolutional kernels, as shown in Fig 2. However, increasing the receptive field to improve feature extraction accuracy also results in exponential growth in the number of model parameters. To address this issue, we adopt the network optimization strategy of Inception V3 [27] and employ a composite convolutional-kernel substitution scheme. Specifically, a single 5 × 5 convolution is decomposed into two sequential 3 × 3 convolutional layers, whereas a single 7 × 7 convolution is decomposed into a cascaded structure comprising three 3 × 3 convolutional layers. This decomposition reduces the number of model parameters by approximately 61% (5 × 5 → 2 × 3 × 3 and 7 × 7 → 3 × 3 × 3) while preserving the equivalent receptive fields and substantially reducing computational complexity. In addition, a parameterized PReLU activation function is introduced into the convolutional blocks. By retaining responses in the negative-value range, PReLU enhances the nonlinear representational capacity of the network and consequently improves its discriminative performance.

The proposed multi-receptive-field feature extraction module adopts a unified architecture in which each receptive-field branch consists of three sequentially connected convolutional blocks. Each block contains a convolutional layer, a batch normalization layer, and a PReLU activation layer. To ensure consistency in feature representation, all convolutional layers contain 32 channels and use a fixed stride of 1, while zero padding preserves the spatial resolution of the feature maps and prevents spatial information loss during feature extraction. The module further incorporates the dense connectivity mechanism of DenseNet [28]. By establishing feedforward connections between each layer and all preceding layers, this mechanism produces exponential growth in the number of feature propagation paths. This architecture allows the network to capture richer semantic information, extract strongly complementary multi-scale feature representations from the source images, and provide high-quality feature inputs for the subsequent fusion stage.

#### Feature generator architecture.

The feature generator is a key component of the deep feature fusion process. It refines the cascaded multi-receptive-field deep features and improves the accuracy of feature representations through feature reconstruction and enhancement. The module comprises three core components: a 3 × 3 convolutional layer with a stride of 1, a batch normalization layer, and a PReLU activation function. This lightweight architecture maintains computational efficiency while facilitating the further extraction of multi-scale feature information and improving feature fidelity.

#### Decoder architecture.

The decoder constitutes the final stage of fused-image generation. Its inputs are the multi-scale features produced by the feature generator, which are integrated to reconstruct a fused image containing comprehensive multimodal information. The decoder adopts a five-layer progressive convolutional architecture. The first convolutional block consists of a 3 × 3 convolutional layer with 32 output channels followed by a PReLU activation function. The second to fourth blocks each contain a 3 × 3 convolutional layer, a batch normalization layer, and a PReLU activation function, with output channel numbers of 32, 32, and 16, respectively. The final block employs a 3 × 3 convolutional layer with one output channel, followed by a Tanh activation function for output normalization. In particular, skip connections directly link the first three convolutional layers of the decoder to the multi-receptive-field deep attention maps produced by the encoder. These connections compensate for semantic information attenuation during deep convolution and strengthen cross-layer feature interaction and fusion. Except for the output layer, which uses the Tanh activation function, the corresponding convolutional blocks employ PReLU activations. The adaptive parameter-learning mechanism of PReLU facilitates model convergence and improves the network’s capacity to represent features in complex scenes.

### Dual-channel hybrid attention fusion module (DCAFM)

**Fig 3:**
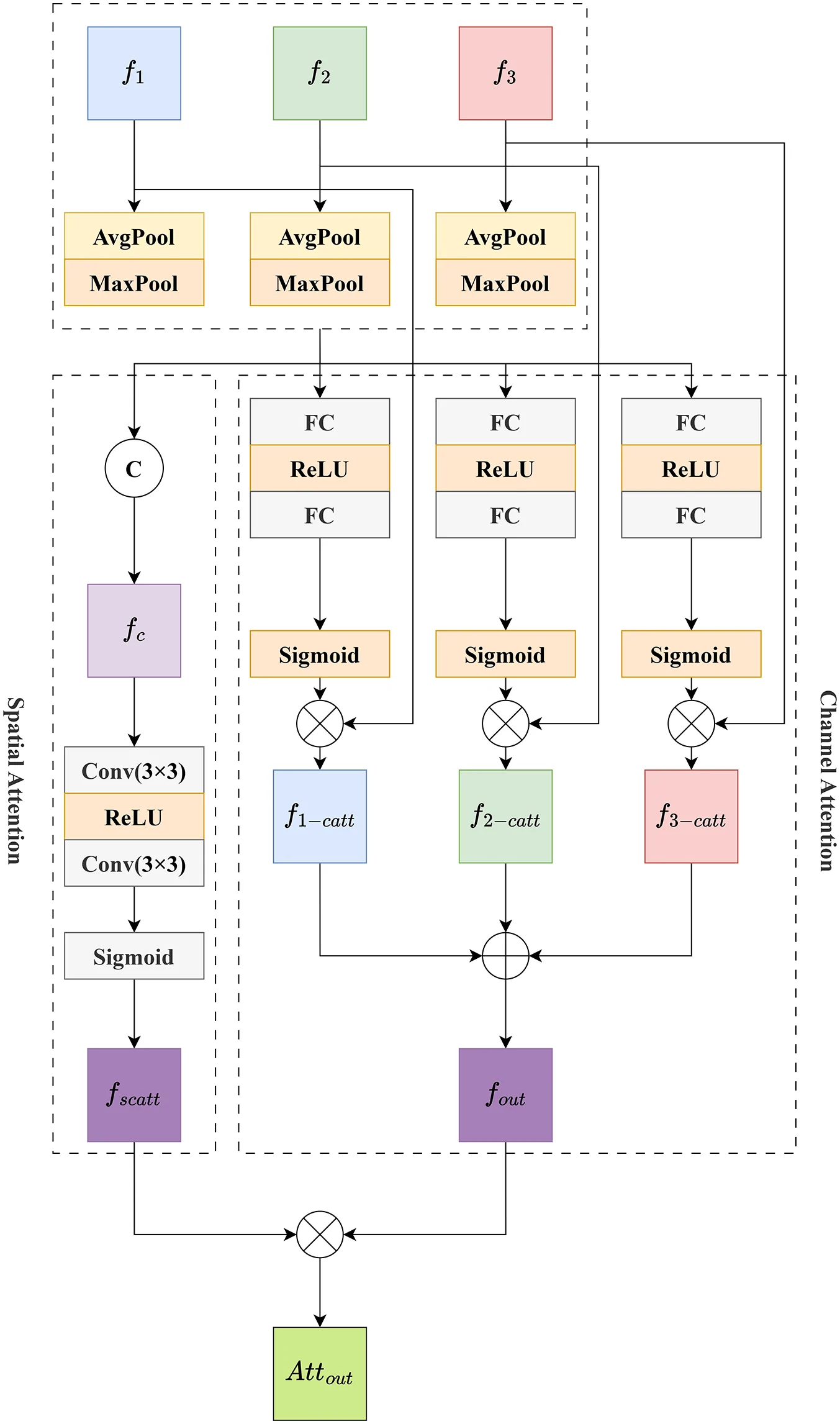
Dual channel hybrid attention fusion module.

The multi-attention mechanism, as the core technology of visual saliency detection, has shown extensive application value in the field of computer vision. Given that infrared and visible light images represent different feature dimensions of the same scene as typical multimodal data, this paper constructs a dual-channel hybrid attention fusion module, as shown in Fig 3 This module synchronously captures salient feature regions from both modalities via its dual-channel attention mechanism. And based on the modal-specific weight allocation strategy, the differentiated representations of the same scene features in different modalities are weighted and enhanced, so that the feature maps generated by the convolutional neural network simultaneously contain the semantic information of the channel dimension and the position information of the spatial dimension. The Attention features from both dimensions carry critical discriminative information. Furthermore, the weight matrices of spatial attention and channel attention are calculated in parallel, and the hierarchical fusion of multi-scale features is carried out based on the receptive field hierarchy. The designed deep attention fusion module adopts a cascading architecture and, through a progressive feature fusion mechanism, enhances the local detail representation while retaining the global semantic association.

1) Fusion of channel attention

First, for the three receptive-field branch features $f_{\text{1}}\in {\mathbb{R}}^{H\times W\times C}$, $f_{\text{2}}\in {\mathbb{R}}^{H\times W\times C}$, and $f_{\text{3}}\in {\mathbb{R}}^{H\times W\times C}$, global average pooling (GAP) and global max pooling (GMP) are applied in parallel over the spatial dimensions H×W, producing two channel feature vectors of size 1×1×C.

The resulting feature vectors are subsequently passed through a two-layer fully connected network for nonlinear transformation, with a ReLU activation function used to enhance their representational capacity. A Sigmoid activation function then normalizes the transformed feature vectors to generate the channel attention weight matrix $W^{c}\in {\mathbb{R}}^{C\times 1\times 1}$. Finally, the original branch features are reweighted using the corresponding channel attention weights, and the weighted outputs of the three receptive-field branches are summed to obtain the channel-enhanced feature representation $f_{\text{out}}^{c}$, which is defined as follows:


$$\begin{array}{c} f_{\text{out}}^{c}=\sum_{i=1}^{\text{3}}\sigma \left(D_{\text{2}}\left(D_{\text{1}}\left(\text{avgp}\left(f_{i}^{c}\right)\right)\right)\right)\times f_{i}^{c} \end{array}$$ (2)

Where i=1,2,3; $f_{\text{1}}^{c}$, $f_{\text{2}}^{c}$, and $f_{\text{3}}^{c}$ denote the channel features extracted by the convolutional branches with receptive fields of 3×3, 5×5, and 7×7, respectively; $D_{\text{1}}$ and $D_{\text{2}}$ denote the two fully connected operations; avgp represents global average pooling; σ(·) denotes the Sigmoid activation function; and H, W, and C represent the height, width, and number of channels of the feature map, respectively.

2) The integration of spatial attention

First, for the three receptive-field branch features $f_{\text{1}}\in {\mathbb{R}}^{H\times W\times C}$, $f_{\text{2}}\in {\mathbb{R}}^{H\times W\times C}$, and $f_{\text{3}}\in {\mathbb{R}}^{H\times W\times C}$, global average pooling (GAP) and global max pooling (GMP) are applied in parallel along the channel dimension, producing three feature maps of size H×W×1. These feature maps are subsequently concatenated along the channel dimension and transformed by a convolutional neural network comprising two 3×3 convolutional layers. A ReLU activation function is used to enhance the nonlinear representational capacity of the network. The intermediate feature map is then normalized using a Sigmoid activation function to generate the spatial attention weight matrix $W^{s}\in {\mathbb{R}}^{H\times W\times 1}$. Finally, the spatial attention weight matrix is combined with the channel-attention-fused feature $f_{\text{out}}^{c}$ through weighted fusion to obtain the deep attention feature map $Att_{\text{out}}^{c}$, which is defined as follows:


$$\begin{array}{c} Att_{\text{out}}^{c}=\sigma \left[Conv_{\text{2}}\left(Conv_{\text{1}}\left(C\left(\text{avgp}\left(f_{i}^{c}\right)\right)\right)\right)\right]\times f_{\text{out}}^{c} \end{array}$$ (3)

Where i=1,2,3; $f_{\text{1}}^{c},f_{\text{2}}^{c},$ and $f_{\text{3}}^{c}$ denote the channel features extracted by the convolutional branches with receptive fields of 3×3, 5×5, and 7×7, respectively; C(·) denotes concatenation along the channel dimension; $Conv_{\text{1}}$ and $Conv_{\text{2}}$ represent two sequential convolution operations with 3×3 kernels; avgp represents global average pooling; σ(·) denotes the Sigmoid activation function; and H,W, and C represent the height, width, and number of channels of the feature map, respectively.

#### Discriminator network architecture.

For the multimodal attribute fusion task of infrared and visible image fusion, the two source modalities exhibit inherent differences while providing complementary information. Accordingly, this section constructs a dual-discriminator architecture comprising an infrared discriminator ($D_{ir}$) and a visible light discriminator ($D_{vis}$). Through joint optimization, the architecture identifies distributional differences between the generated image and the corresponding modality-specific source images while maintaining a relative balance between their multimodal attributes. Specifically, the infrared discriminator ($D_{ir}$) constrains the fused image to retain intensity characteristics from the infrared modality, whereas the visible light discriminator ($D_{vis}$) enhances the representation of detailed textures from the visible modality. These differentiated constraints effectively mitigate intermodal information conflicts during multimodal fusion and facilitate the integration of complementary features.

During network training, a dynamic balance must be maintained among the generator (G), the infrared discriminator ($D_{ir}$), and the visible light discriminator ($D_{vis}$). If any of these networks is optimized considerably faster than the others, the optimization processes of the remaining two networks may be suppressed. The dominant network may then impose stronger modality-specific constraints, biasing the fused output towards the corresponding modality and weakening or even offsetting the effects of the other networks. Such training imbalance can distort the information-retention mechanism of the fused image and consequently degrade its overall performance. Establishing an effective network-balancing strategy is therefore essential for the collaborative optimization of multimodal features.

**Fig 4:**
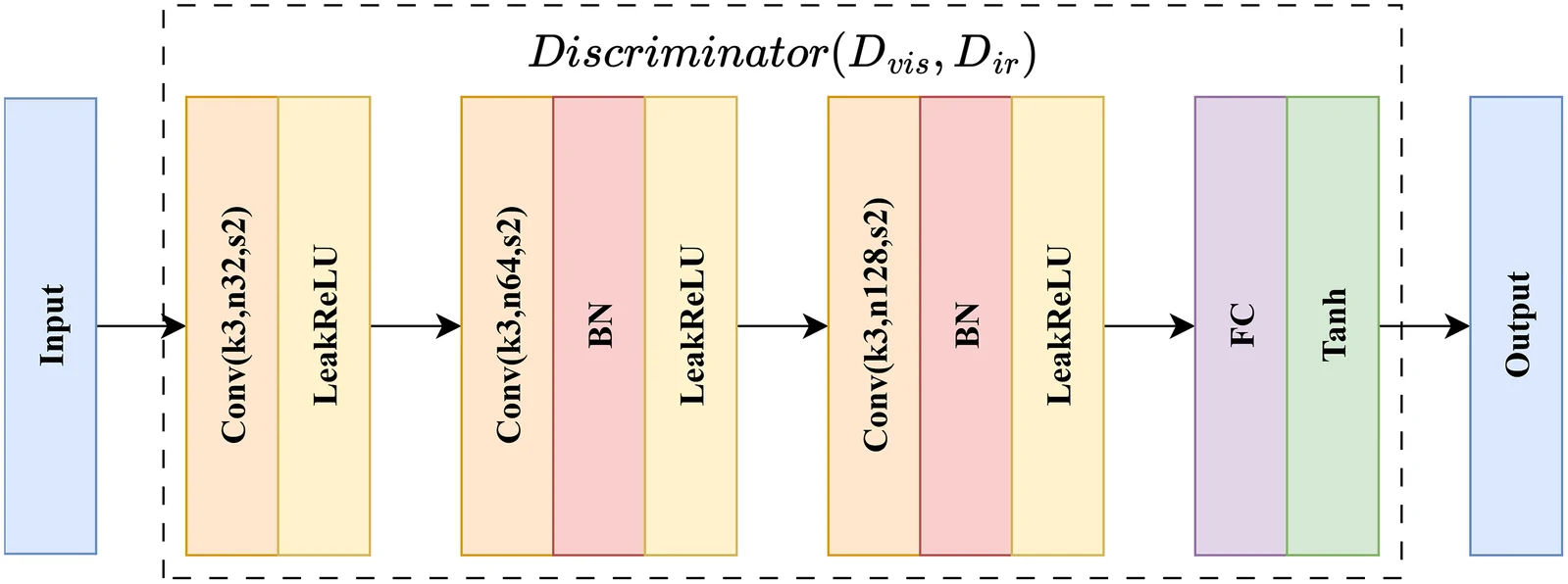
Network framework of discriminator.

The proposed DHAFGan constrains the balance of the training process through the coordinated design of its network architecture and loss functions. Because each discriminator operates as a binary classifier and has lower structural complexity than the generator, its primary function is to determine whether an input image is real or generated. Therefore, a symmetric Siamese network architecture is adopted to construct the dual discriminators, as illustrated in Fig 4. The infrared discriminator ($D_{ir}$) and visible light discriminator ($D_{vis}$) share the same network architecture but have independent parameters. Each discriminator consists of three cascaded convolutional feature extraction blocks followed by a fully connected output layer. Specifically, the first convolutional block comprises a 3 × 3 convolutional layer and a LeakyReLU activation function, whereas the second and third blocks each comprise a 3 × 3 convolutional layer, a batch normalization (BN) layer, and a LeakyReLU activation function. After convolutional feature extraction, the fully connected layer performs the final discrimination, and a Tanh activation function generates the output. All three convolutional layers use 3 × 3 kernels with a stride of 2, allowing the spatial dimensions of the feature maps to decrease progressively during discrimination. The channel numbers are set to 32, 64, and 128 with increasing network depth, thereby forming a hierarchical feature-abstraction structure that progresses from low-level texture features to high-level discriminative semantic representations.

#### Loss function.

The loss function is essential for maintaining the dynamic balance between the generator and the discriminators. During adversarial training, the generator and discriminators are jointly optimized to achieve balanced retention of information from the source images, while the generator is required to produce outputs whose data distribution is consistent with that of the source images. Accordingly, the proposed loss function imposes constraints from two perspectives: spatial structural similarity and semantic-content fidelity. At the structural level, the adversarial loss encourages the statistical characteristics of the generated image to approximate the data distributions of the real source images. At the content level, a multi-scale feature loss is introduced to preserve essential semantic information from the source images. This dual-constraint mechanism improves the realism and information completeness of the fused image while reducing the ability of the discriminators to distinguish the generated image from the real source images, thereby facilitating the convergence of adversarial training. The optimization objective of DHAFGan consists of the generator loss ${ℒ}_{G}$ and discriminator loss ${ℒ}_{D}$. The generator loss comprises the adversarial loss ${ℒ}_{adv}$ and the weighted content loss ${ℒ}_{c}$, as defined below:


$$\begin{array}{c} \left\{ \begin{array}{c} @l@min{ℒ}_{G}={ℒ}_{adv}+\lambda {ℒ}_{c}\\ {ℒ}_{c}=\xi_{\text{1}}{ℒ}_{ssim}+\xi_{\text{2}}{ℒ}_{text}+\xi_{\text{3}}\\ {ℒ}_{grad},min{ℒ}_{D}=\left\{ \begin{array}{c} @l@{ℒ}_{D_{ir}}\\ {ℒ}_{D_{vis}} \end{array}\right. \end{array}\right. \end{array}$$ (4)

Where ${ℒ}_{G}$ denotes the generator loss; ${ℒ}_{adv}$ is the generator adversarial loss; ${ℒ}_{c}$ represents the content feature loss; λ, $\xi_{\text{1}}$, $\xi_{\text{2}}$, and $\xi_{\text{3}}$ are hyperparameters; ${ℒ}_{ssim}$ denotes the structural similarity loss; ${ℒ}_{text}$ is the texture loss; ${ℒ}_{grad}$ is the gradient loss; ${ℒ}_{D}$ represents the discriminator loss; ${ℒ}_{D_{ir}}$ is the infrared discriminator loss; and ${ℒ}_{D_{vis}}$ is the visible light discriminator loss.

#### Generator adversarial loss$L_{adv}$.

The generator adversarial loss comprises two components. The infrared-discriminator-based adversarial loss constrains the authenticity of the fusion result in the infrared feature space, whereas the visible-light-discriminator-based adversarial loss maintains the visual fidelity of the generated image in the visible modality. Through differentiated constraints, this dual-branch adversarial loss mechanism enables the collaborative optimization of the multimodal feature space, allowing the fused image to accurately represent infrared target features while retaining scene details from the visible image. The generator adversarial loss is defined as follows:


$$\begin{array}{c} {ℒ}_{adv}=-{𝔼}_{I_{f}\;P_{G}}\left[D_{\text{ir}}\left(I_{f}\right)\right]-{𝔼}_{I_{f}\;P_{G}}\left[D_{\text{vis}}\left(I_{f}\right)\right] \end{array}$$ (5)

Where $I_{f}$ denotes the fused-image sample produced by the generator, $P_{G}$ represents the data distribution of the fused image, and $D_{ir}$ and $D_{vis}$ denote the infrared discriminator and visible light discriminator, respectively.

The structural similarity loss ${ℒ}_{ssim}$ is defined as follows:


$$\begin{array}{c} {ℒ}_{ssim}=\theta_{\text{1}}\left[1-ssim\left(I_{f},I_{\text{1}}\right)\right]+\theta_{\text{2}}\left[1-ssim\left(I_{f},I_{\text{2}}\right)\right] \end{array}$$ (6)

Where $\theta_{\text{1}}$ and $\theta_{\text{2}}$ are hyperparameters, both set to 0.5; $I_{\text{1}}$, $I_{\text{2}}$, and $I_{f}$ denote the infrared image, visible image, and fused image, respectively; and ssim(·) denotes the structural similarity operation.

The texture loss ${ℒ}_{text}$ is defined as follows:


$$\begin{array}{c} {ℒ}_{\text{text}}=\frac{∥|\nabla I_{f}|-max\left(\right|\nabla I_{\text{1}}\left|,\right|\nabla I_{\text{2}}\left|\right){∥}_{\text{1}}}{HW} \end{array}$$ (7)

Where ∇ denotes the Sobel gradient operator used to extract texture information; |·| represents the element-wise absolute-value operation; $∥\cdot {∥}_{\text{1}}$ denotes the matrix L1 norm; and H and W represent the image height and width, respectively.

The gradient loss ${ℒ}_{grad}$ is defined as follows:


$$\begin{array}{c} \begin{array}{c} {ℒ}_{grad}={ℒ}_{grad_{main}}+{ℒ}_{grad_{aux}}\\ =\delta_{\text{1}}{\|I_{f}-I_{\text{i}r}\|}_{F}^{\text{2}}+\delta_{\text{2}}{\|\nabla I_{f}-\nabla I_{vis}\|}_{F}^{\text{2}}\\ +\delta_{\text{3}}{\|I_{f}-I_{vis}\|}_{F}^{\text{2}}+\delta_{\text{4}}{\|\nabla I_{f}-\nabla I_{ir}\|}_{F}^{\text{2}} \end{array} \end{array}$$ (8)

Where ${ℒ}_{grad_{main}}$ and ${ℒ}_{grad_{aux}}$ denote the primary and auxiliary gradient losses, respectively; $\delta_{\text{1}},\delta_{\text{2}},\delta_{\text{3}},\delta_{\text{4}}$ are hyperparameters; $I_{f}$, $I_{\text{ir}}$, and $I_{vis}$ denote the fused image, infrared source image, and visible source image, respectively; ∇ represents the Laplacian operator used to extract edge information; and $∥\cdot {∥}_{F}^{\text{2}}$ denotes the squared Frobenius norm.

#### Discriminator loss $L_{𝐃}$.

The proposed method adopts a dual-discriminator architecture with two independently optimized objectives: the infrared discrimination loss and the visible light discrimination loss. The two discriminator branches impose differentiated constraints according to the modality differences between the infrared and visible source images, enabling the fused image to retain modality-specific information from both sources. Specifically, the visible light discriminator focuses on preserving high-frequency information, including scene textures and fine details, whereas the infrared discriminator primarily maintains target contours, intensity information, and contrast characteristics. Compared with a single-discriminator architecture, the dual-discriminator design strengthens feature complementarity between the two modalities and facilitates the effective integration of cross-modal information, thereby improving the representational capacity of the fusion result in a multidimensional feature space. The infrared and visible light discrimination losses are defined as follows:


$$\begin{array}{c} \begin{array}{c} {ℒ}_{D_{ir}}=-E_{x\;P_{ir}}\left[D_{ir}\left(x\right)\right]+E_{I_{f}\;P_{G}}\left[D_{ir}\left(I_{f}\right)\right]\\ +\phi_{\text{1}}E_{\tilde{x}\;p_{penalty}}\left[{\left(∥\nabla_{\tilde{x}}D_{ir}\left(\tilde{x}\right){∥}_{\text{2}}-1\right)}^{\text{2}}\right] \end{array} \end{array}$$ (9)

$$\begin{array}{c} \begin{array}{c} {ℒ}_{D_{vis}}=-E_{x\;P_{vis}}\left[D_{vis}\left(x\right)\right]+E_{I_{f}\;P_{G}}\left[D_{vis}\left(I_{f}\right)\right]\\ +\phi_{\text{2}}E_{\tilde{x}\;p_{penalty}}\left[{\left(∥\nabla_{\tilde{x}}D_{vis}\left(\tilde{x}\right){∥}_{\text{2}}-1\right)}^{\text{2}}\right] \end{array} \end{array}$$ (10)

Where $D_{ir}$ and $D_{vis}$ denote the infrared discriminator and visible light discriminator, respectively; x represents a real source-image sample, and $I_{f}$ denotes a fused-image sample produced by the generator; $P_{ir}$ and $P_{vis}$ represent the data distributions of the infrared and visible source images, respectively, whereas $P_{G}$ denotes the data distribution of the fused images; $\tilde{x}$ represents a sample drawn uniformly from the continuous region between the real and generated sample spaces; and $\phi_{\text{1}}$ and $\phi_{\text{2}}$ are hyperparameters used to balance the corresponding discriminator losses.

## Experimental

### Experimental setup

The experiments were conducted on Ubuntu 18.04 using an NVIDIA GeForce RTX 3060 GPU with 12 GB of memory and the PyTorch deep learning framework. The algorithm parameters were configured as follows: the hyperparameters in the discriminator loss function were set to $\delta_{\text{1}}=1$, $\delta_{\text{2}}=1$, $\delta_{\text{3}}=3$, and $\delta_{\text{4}}=0.2$; the weight parameter in the generator loss function was set to λ=9; and the three weight parameters in the content loss function were set to $\xi_{\text{1}}=9$, $\xi_{\text{2}}=9$, and $\xi_{\text{3}}=9$. The initial learning rate was set to 0.0001, with a learning-rate decay factor of 0.9. The model was trained for 100 epochs with a batch size of 8, and the network parameters were updated using the Adam optimizer. In addition, to alleviate the potential overdependence of generator training on the discriminators, the update-frequency ratio between the generator and the discriminators was set to 2:4.

To evaluate the performance of the proposed algorithm, experiments were conducted using the DroneVehicle dataset. Released by Tianjin University, DroneVehicle [29] is a large-scale vehicle dataset collected from unmanned aerial vehicles. It contains 28,439 registered pairs of RGB/visible and infrared images, corresponding to a total of 56,878 individual images. The dataset covers a variety of road scenes, including highways, communities, and residential areas, as well as different imaging conditions, such as daytime and nighttime. The target categories include vans, trucks, and vehicles transporting hazardous materials. Because the original images contain a 100-pixel-wide white border around their edges, all images were batch-cropped before model training to remove these borders and prevent them from interfering with feature learning and image fusion performance.

**Fig 5:**
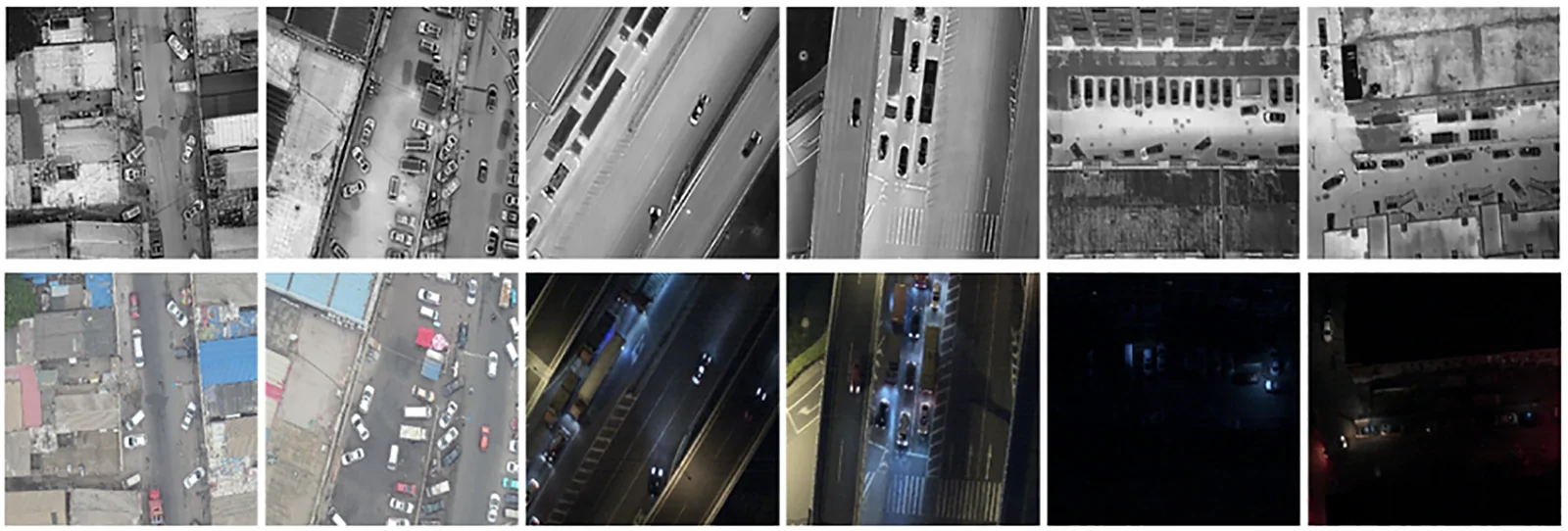
Some typical image pairs in the DroneVehicle dataset. Note:The images are sourced from the DroneVehicle dataset, available at https://github.com/VisDrone/DroneVehicle.

Fig 5 shows several representative image pairs from the DroneVehicle dataset. In the main experiments, 30 image pairs were randomly selected from the DroneVehicle dataset to construct the test set, while the remaining 28,409 image pairs were used for model training. The subjective and objective comparisons of multiple fusion algorithms were then conducted on this test set. Furthermore, this study conducted an additional robustness analysis under different test set sizes. In this supplementary experiment, 100 image pairs were first randomly selected from the DroneVehicle dataset as an independent test pool, while the remaining 28,339 image pairs were used for model training. Subsequently, test subsets containing 30, 50, 80, and 100 image pairs were constructed from this independent test pool to analyze the performance stability of the proposed method under different test set sizes.

### Evaluation index

This paper mainly evaluates the fusion results from both subjective and objective aspects. Subjective evaluation involves human observers assessing the fused images based on criteria such as target distinctness, overall clarity, richness of texture details, and visual naturalness. The overall quality is then rated using a qualitative scale (e.g., “excellent,” “good,” “fair,” “poor”). Different from subjective evaluation methods, objective evaluation methods mainly quantitatively evaluate image quality through statistical calculations of information such as the gradient, intensity, and contrast of the target object in the fused image. Since a ground-truth fused image is unavailable for real-world fusion tasks, objective evaluation relies on the characteristics of the source images. We adopt several widely-used metrics: The Average Gradient (AG) [30] measures the ability of the fused image to preserve detailed texture information, which is prominent in visible images. The Standard Deviation (SD) [31] was adopted to measure the gray level and contrast of the salient regions in the fused image, and Mutual Information (MI) [32] was used to determine whether the fused image evenly retains the salient features in the source image. Finally, the peak signal-to-noise ratio (PSNR) [33] was used to measure the difference between the fused image and the ideal image.

#### Average gradient (AG).

The average gradient measures the ability of the fused image to retain the texture detail information in the source image by statistically fusing the gradient information in the horizontal and vertical directions of the image and then calculating the mean value. The larger its value is, the more abundant the texture details of the fused image are retained. It is defined as follows:


$$\begin{array}{c} \begin{array}{c} AG=\frac{\text{1}}{M\times W}\sum_{i=\text{1}}^{M-\text{1}}\sum_{j=\text{1}}^{W-\text{1}}\sqrt{\frac{\text{1}}{\text{2}}\left(f_{i}^{\text{2}}(i,j)+f_{j}^{\text{2}}(i,j)\right)} \end{array} \end{array}$$ (11)

Where M and W are the height and width of the fused image; f(i,j) is the gray value magnitude of the fused image at position; $f_{i}^{\text{2}}(i,j)$ is the gradient information of the fused image in the horizontal direction, and $f_{j}^{\text{2}}(i,j)$ is the gradient information of the fused image in the vertical direction.

#### Standard deviation (SD).

The standard deviation represents the fluctuation between pixel values by statistically analyzing the contrast and the dispersion degree of distribution in the fused image. The larger its value is, the more obvious the contrast feature in the infrared part of the fused image is, and the clearer the detailed information in the visible light part is. It is defined as follows:


$$\begin{array}{c} SD=\sqrt{\frac{\text{1}}{M\times W}\sum_{i=1}^{M}\sum_{j=1}^{W}{\left(F\left(i,j\right)-\mu \right)}^{\text{2}}} \end{array}$$ (12)

where F(i,j) denotes the grayscale value of the fused image at pixel position (i,j); M and W represent the height and width of the fused image, respectively; μ denotes the mean grayscale value of the fused image; and $\frac{\text{1}}{\left(M\times W\right)}$ is the normalization factor used to calculate the mean of the squared grayscale deviations over all pixels, thereby ensuring that the metric conforms to the definition of standard deviation.

#### Mutual information (MI).

Mutual information measures the degree of information sharing between the source image and the fused image by calculating the normalized histogram. The larger the value, the better the fused image retains the object information in the salient regions of the source image, and there is less secondary information loss in the non-salient regions. It is defined as follows:


$$\begin{array}{c} \begin{array}{c} MI(I,J)=\frac{\text{1}}{\text{2}}\left(MI_{A,F}+MI_{B,F}\right)=\frac{\text{1}}{\text{2}}\left(\begin{array}{c} \sum_{i=\text{1}}^{L-\text{1}}\sum_{j=\text{1}}^{L-\text{1}}q_{A,F}(i,j)\cdot {log}_{\text{2}}\frac{q_{A,F}(i,j)}{q_{A}(i)q_{F}(j)}\\ +\sum_{i=\text{1}}^{L-\text{1}}\sum_{j=\text{1}}^{L-\text{1}}q_{B,F}(i,j)\cdot {log}_{\text{2}}\frac{q_{B,F}(i,j)}{q_{B}(i)q_{F}(j)} \end{array}\right) \end{array} \end{array}$$ (13)

Where L is the gray scale number of the fused image; $MI_{\text{A},\text{F}}$ and $MI_{\text{B},\text{F}}$ are the normalized calculation of the single-source image and the fused image respectively; $q_{A}(i)$, $q_{B}(i)$, and $q_{F}(j)$ are the normalized histogram distribution maps of the source image A, source image B, and the fused image F respectively.

#### Peak signal-to-noise ratio (PSNR).

The peak signal-to-noise ratio is calculated as the ratio of the maximum pixel value of the fused image to the mean square of the pixel values between the fused image and the ideal image to measure the distortion between the source image and the fused result image. The larger the value, the less distortion of the fused image. It is defined as follows:


$$\begin{array}{c} \begin{array}{c} PSNR=\text{1}\text{0}\cdot {\text{log}}_{\text{1}\text{0}}\left(\frac{MAX_{I}^{\text{2}}}{MSE}\right) \end{array} \end{array}$$ (14)

$$\begin{array}{c} MSE=\frac{\text{1}}{M\times W}\sum_{i=\text{1}}^{M}\sum_{j=\text{1}}^{W}{\left[I(i,j)-K(i,j)\right]}^{\text{2}} \end{array}$$ (15)

Where MSE is the mean square deviation of the overall pixel values between the source image and the fused image; *MAX*_*I*_ is the maximum possible pixel value of the fused image.

## Analysis and discussion

### Ablation experiment

**Fig 6:**
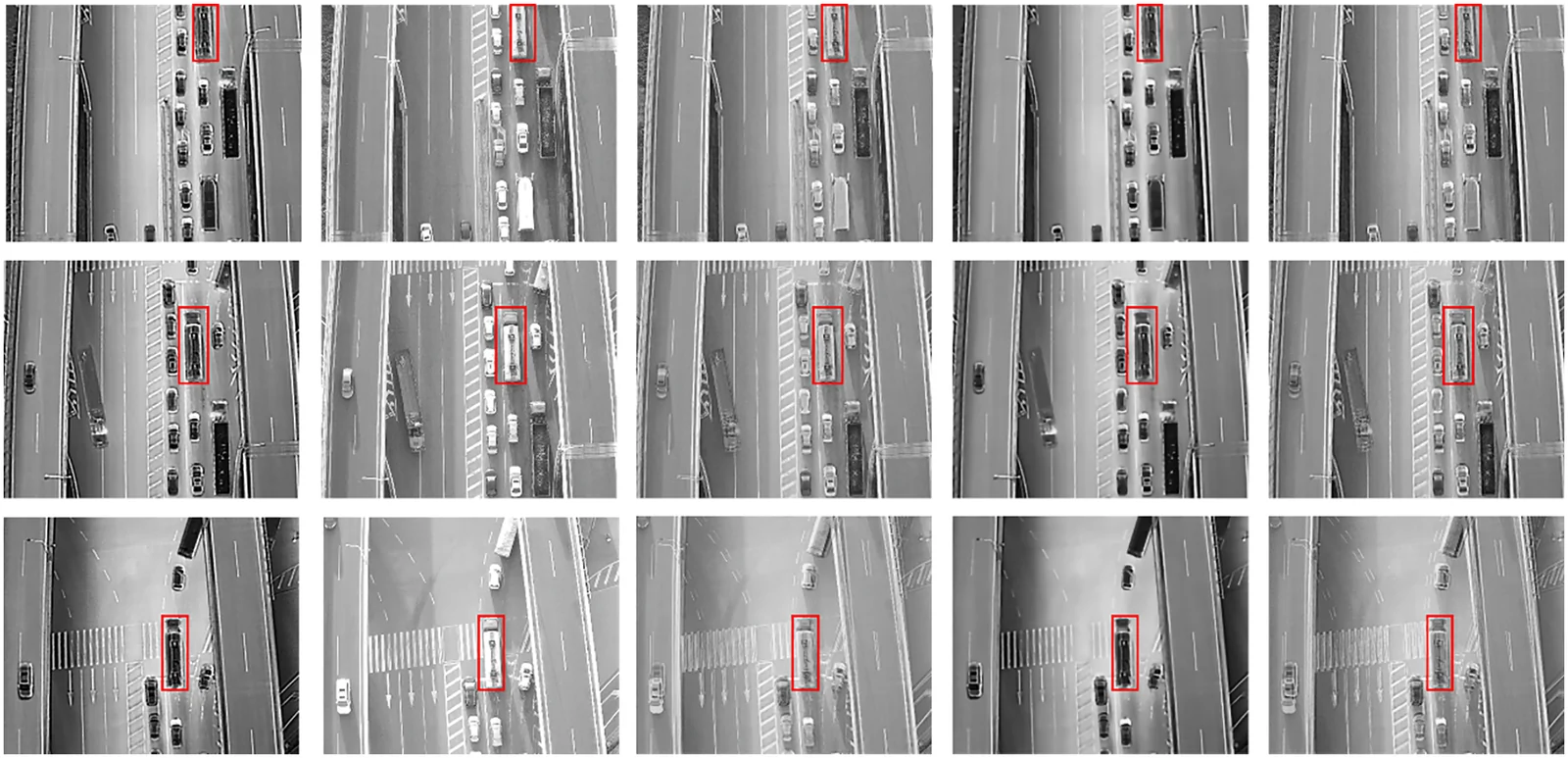
Visualization result graph of the ablation experiment Note:The images are sourced from the DroneVehicle dataset, available at https://github.com/VisDrone/DroneVehicle. (a) Infrared image, (b) Visible light image, (c) w/o M1, (d) w/o M2, (e) DHAFGan.

This section evaluates the contributions of the shallow-deep feature extraction module (M1) and the dual-channel hybrid attention fusion module (M2) through three ablation configurations. The qualitative and quantitative results are presented in Fig 6 and Table 1, respectively, with the best results highlighted in bold. Replacing M1 with stacked conventional convolutional blocks (w/o M1) reduced the mean SD, AG, MI, and PSNR from 9.3541, 3.9854, 2.4321, and 65.7852 to 7.5459, 2.9940, 2.0543, and 62.7350 dB, respectively. These decreases indicate that the multi-receptive-field design of SDFE is important for extracting infrared target contrast while preserving texture details from visible images. Removing M2 (w/o M2) likewise reduced the four mean metrics to 8.4375, 2.8490, 1.8572, and 60.2540, respectively, supporting the role of the dual-channel attention mechanism in enhancing target saliency and preserving structural information. For all four metrics, the bootstrap 95% confidence intervals of the complete DHAFGan configuration did not overlap those of either ablated configuration. Overall, these results support the complementary contributions and design rationale of M1 and M2 in improving fusion performance.

**Table 1:** Quantitative Comparison of Ablation Experiments.

Method	SD(Mean±image s.d.)	SD 95% CI	AG(Mean±image s.d.)	AG 95% CI	MI(Mean±image s.d.)	MI 95% CI	PSNR(Mean±image s.d.)	PSNR 95% CI
w/o M1	7.5459 ± 1.2843	7.0927–7.9993	2.9940 ± 0.4728	2.8300–3.1625	2.0543 ± 0.3917	1.9192–2.1944	62.7350 ± 2.3374	61.9210–63.5872
w/o M2	8.4375 ± 1.0976	8.0572–8.8284	2.8490 ± 0.5581	2.6498–3.0342	1.8572 ± 0.4386	1.7009–2.0104	60.2540 ± 2.4629	59.3783–61.1087
DHAFGan	9.3541 ± 1.0365	8.9815–9.7096	3.9854 ± 0.4939	3.8105–4.1561	2.4321 ± 0.3301	2.3159–2.5490	65.7852 ± 2.0150	65.0786–66.4938

Note: Data are presented as mean ± between-image standard deviation, with non-parametric bootstrap 95% confidence intervals. A total of 30 test image pairs are included. Bold values indicate the optimal results.

### Comparative experiment

#### Subjective qualitative evaluation.

**Fig 7:**
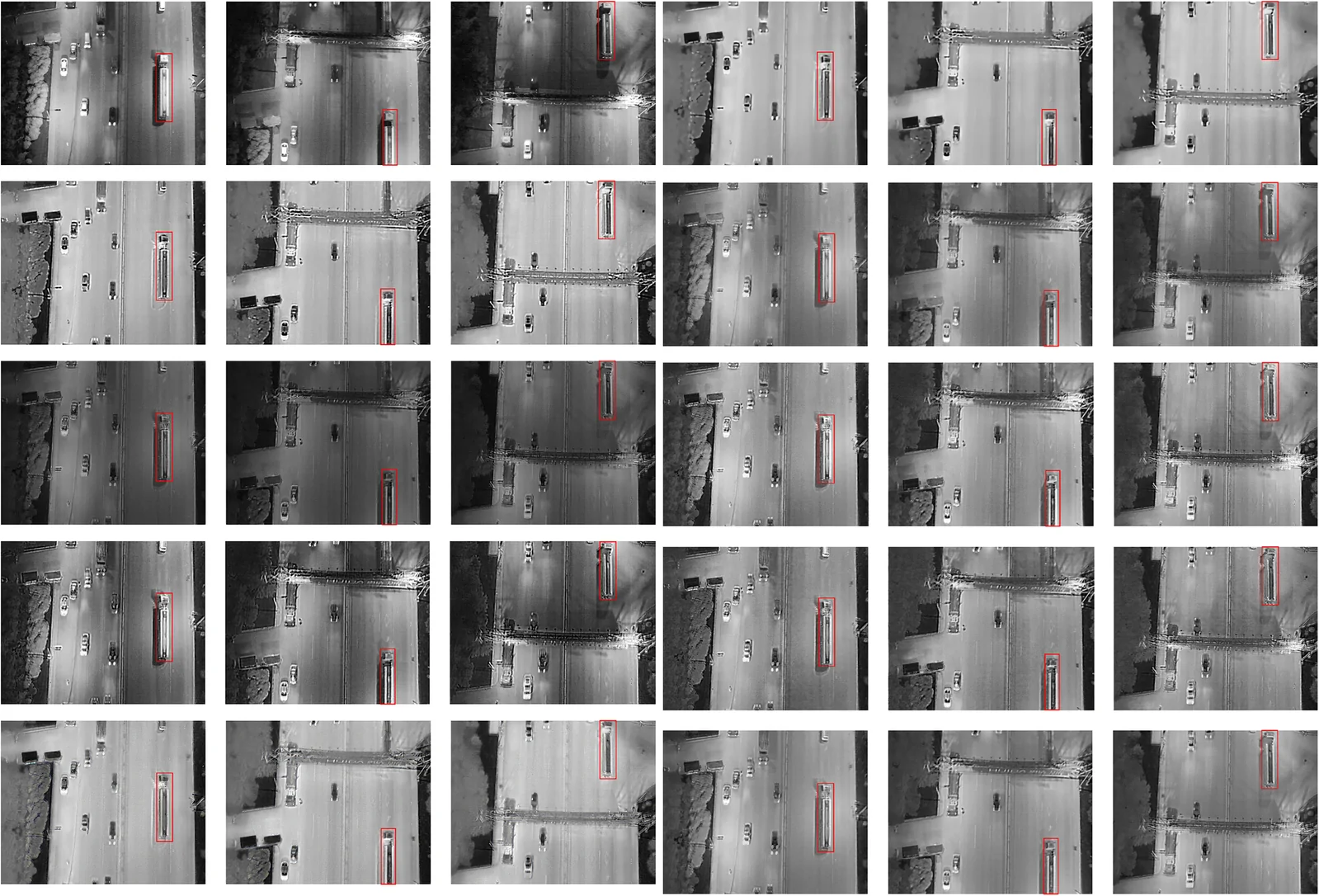
A comparison chart of the fusion results of the DroneVehicle dataset. Note: The images are sourced from the DroneVehicle dataset, available at https://github.com/VisDrone/DroneVehicle. (a) Visible image, (b) Infrared image, (c) ADF, (d) GFF, (e) GTF, (f) RP, (g) CNN, (h) FusionGAN, (i) GANMcC, (j) DHAFGan.

To systematically validate the performance advantages of the proposed algorithm, this section conducts a qualitative comparative analysis on three sets of representative fusion images selected from the test results on the DroneVehicle dataset. The experimental results are presented in Fig 7. The figure displays the fusion results of visible light images, infrared images, and eight different fusion algorithms, including Anisotropic Diffusion Fusion (ADF), Guided Filtering Fusion (GFF), Gradient Transfer Fusion (GTF), Ratio Pyramid (RP), Convolutional Neural Networks (CNN), Fusion Generative Adversarial Network (FusionGAN), Generative Adversarial Network with Multi-Class Constraints (GANMcC), and the proposed Dense Hybrid Attention Fusion Generative Adversarial Network (DHAFGan). As shown in Fig 7, significant differences can be observed among the algorithms in terms of information retention, contrast enhancement, texture sharpness, and visual naturalness. The detailed analysis is as follows:

The fusion results of the ADF algorithm generally exhibit low overall brightness and insufficient image contrast, leading to poor distinction between targets and the background. Edge information in certain regions is blurred, particularly in details such as vehicle contours and road markings, resulting in an ineffective preservation of texture features from the visible light image.

Although the GFF algorithm can preserve the intensity information of the infrared image to some extent, the fusion images exhibit noticeable edge blurring. Texture information of vehicle targets is almost entirely lost, and background details are poorly represented, resulting in an overly smooth overall visual effect lacking in layering.

The GTF algorithm demonstrates prominent performance in enhancing target saliency and effectively preserves intensity information from the infrared image. However, excessive sharpening of moving vehicles leads to halo effects and artifacts in certain areas, causing visual distortion and compromising the naturalness of the image.

The fusion results of the RP algorithm achieve a relatively balanced performance in overall contrast and brightness. However, it still falls short in preserving detailed textures, with over-smoothing observed in certain regions, particularly in road textures and vehicle contours, leading to noticeable information loss.

Compared with traditional methods, the CNN algorithm performs better in balancing information, effectively retaining texture details from the visible light image and intensity information from the infrared image. The fusion results are visually natural with high edge sharpness, though slight overexposure occurs in some highlight regions.

The FusionGAN algorithm preserves target intensity information from the infrared image relatively well but inadequately retains texture details from the visible light image, resulting in blurring and artifacts in some areas. The overall visual effect is somewhat dark, and the visual naturalness requires improvement.

The GANMcC algorithm exhibits moderate performance in integrating multi-modal information. The fusion images suffer from severe loss of background texture details and information loss in certain target regions, leading to poor overall visual quality and failing to meet the requirements of high-quality image fusion.

Compared with the aforementioned seven algorithms, the proposed DHAFGan algorithm demonstrates significant advantages in subjective visual performance. Specifically, DHAFGan completely preserves the structural features of the iron framework of occluded vehicles from the visible light image while effectively extracting and integrating salient target information from the infrared image, achieving a balanced representation of multi-modal features. The fusion results exhibit high visual similarity to the source images, without noticeable distortion, artifacts, or noise interference. The overall image brightness is moderate, and the contrast is natural, aligning well with human visual perception. In detailed regions such as road markings, vehicle contours, and background buildings, DHAFGan maintains high sharpness and texture integrity, effectively avoiding issues such as excessive sharpening and information loss commonly observed in traditional methods.

In summary, from a subjective visual perspective, the fused images generated by the DHAFGan algorithm achieve a high degree of consistency in apparent quality with visible light images while fully retaining the salient target information from infrared images. The algorithm significantly outperforms the other seven comparative algorithms in terms of information retention, visual naturalness, and texture sharpness, thereby fully validating its superior performance in multi-modal image fusion tasks.

#### Objective quantitative evaluation.

The quantitative comparison results are presented in Table 2, which reports the mean, between-image standard deviation, and nonparametric bootstrap 95% confidence interval for each metric across 30 test image pairs. DHAFGan achieved the highest mean values for SD, MI, and PSNR, reaching 9.3541, 2.4321, and 65.7852, respectively. These results indicate that the proposed method performs strongly in enhancing image contrast, preserving complementary information from the source modalities, and reducing fusion distortion. For AG, DHAFGan ranked second with a mean value of 3.9854, following RP at 4.4125, demonstrating its competitive ability to preserve texture-gradient information. The interval estimates showed partial overlap between DHAFGan and several strong comparison methods for some metrics. Accordingly, the results are best interpreted by jointly considering the mean rankings and interval estimates. Overall, DHAFGan delivered well-balanced performance across the four complementary metrics, demonstrating a consistent leading trend and strong overall competitiveness and further supporting its effectiveness in multimodal image fusion tasks.

**Table 2:** Quantitative comparison results of multiple fusion algorithms in the dronevehicle dataset.

	SD(Mean±image s.d.)	SD 95% CI	AG(Mean±image s.d.)	AG 95% CI	MI(Mean±image s.d.)	MI 95% CI	PSNR(Mean±image s.d.)	PSNR 95% CI
ADF	8.4684 ± 1.1147	8.0810–8.8635	2.9132 ± 0.3532	2.7937–3.0406	1.8536 ± 0.3854	1.7193–1.9868	65.4816 ± 2.1144	64.7478–66.2296
FusionGAN	8.1253 ± 1.1818	7.7085–8.5393	2.2356 ± 0.5410	2.0398–2.4254	2.1873 ± 0.3604	2.0659–2.3166	61.2894 ± 2.0436	60.5944–62.0153
GANMcC	9.1548 ± 1.0004	8.8054–9.4998	1.8732 ± 0.5655	1.6827–2.0781	0.8941 ± 0.3516	0.7752–1.0171	62.6542 ± 2.2796	61.8442–63.4745
GFF	8.4526 ± 1.0634	8.0644–8.8233	3.3561 ± 0.4264	3.2032–3.4998	2.1357 ± 0.3380	2.0123–2.2516	61.3781 ± 2.2808	60.5959–62.1941
GTF	8.3261 ± 0.9057	8.0066–8.6488	2.5943 ± 0.4628	2.4315–2.7594	2.3148 ± 0.3788	2.1879–2.4499	62.9155 ± 1.9434	62.2373–63.5862
CNN	8.7536 ± 1.2199	8.3145–9.1909	3.7634 ± 0.5041	3.5862–3.9417	1.8549 ± 0.4143	1.7064–1.9970	63.5732 ± 2.1817	62.8153–64.3311
RP	8.6951 ± 1.1726	8.2739–9.0993	**4.4125 ± 0.5213**	4.2274–4.5966	1.8261 ± 0.4212	1.6751–1.9697	65.4561 ± 1.6995	64.8546–66.0585
DHAFGan	**9.3541 ± 1.0365**	8.9720–9.7069	3.9854 ± 0.4939	3.8161–4.1626	**2.4321 ± 0.3301**	2.3174–2.5489	**65.7852 ± 2.0150**	65.0765–66.4920

Note: Data are presented as mean ± between-image standard deviation, with non-parametric bootstrap 95% confidence intervals. A total of 30 test image pairs are included. Bold values indicate the optimal results, and underlined values indicate the second-best results.

To further examine the influence of sample size on the experimental results, the number of training image pairs was fixed at 28,339, and test subsets containing 30, 50, 80, and 100 image pairs were evaluated. As shown in Table 3, when the test-set size increased from 30 to 100 image pairs, the mean SD changed from 9.3010 to 8.9689, MI from 2.4257 to 2.3076, and PSNR from 65.8666 to 64.5356, whereas AG remained within the range of 3.8523–3.9848. The between-repeat standard deviations generally decreased with increasing test-set size: from 0.2579 to 0.1517 for SD, from 0.0927 to 0.0672 for MI, and from 0.7385 to 0.4573 for PSNR, while the variability in AG remained low (0.0948–0.1276). These findings indicate that estimates obtained from larger test sets are less sensitive to subset composition and that DHAFGan maintains broadly stable fusion performance across different test-set sizes. Collectively, the results suggest that the performance of DHAFGan does not depend on a particular small test sample and remains stable across the evaluated test-set sizes.

**Table 3:** Robustness analysis of DHAFGan on the DroneVehicle dataset under different test set sizes.

Training pairs	Test pairs	Repeats	SD(Mean±repeat s.d.)	SD 95% CI	AG(Mean±repeat s.d.)	AG 95% CI	MI(Mean±repeat s.d.)	MI 95% CI	PSNR(Mean±repeat s.d.)	PSNR 95% CI
28,339	30	30	9.3010 ± 0.2579	9.2088–9.3894	3.9848 ± 0.1276	3.9405–4.0299	2.4257 ± 0.0927	2.3948–2.4599	65.8666 ± 0.7385	65.6123–66.1309
28,339	50	30	9.2337 ± 0.1986	9.1642–9.3039	3.8523 ± 0.1093	3.8135–3.8895	2.4056 ± 0.0834	2.3757–2.4348	65.2774 ± 0.5912	65.0693–65.4895
28,339	80	30	9.1482 ± 0.1664	9.0907–9.2075	3.8947 ± 0.0948	3.8616–3.9272	2.3231 ± 0.0719	2.2981–2.3485	65.0442 ± 0.4826	64.8759–65.2196
28,339	100	30	8.9689 ± 0.1517	8.9150–9.0211	3.8939 ± 0.1015	3.8586–3.9297	2.3076 ± 0.0672	2.2842–2.3305	64.5356 ± 0.4573	64.3748–64.6930

Note: This table summarizes the supplementary robustness experiment using four prespecified test subsets containing 30, 50, 80, and 100 image pairs. The training set was fixed at 28,339 image pairs, and all test images were drawn from an independent 100-pair test pool that was excluded from model training. Values are reported as the mean ± between-image s.d., with non-parametric bootstrap 95% confidence intervals calculated at the image-pair level. Higher values indicate better fusion performance.

#### Comparison of computational efficiency.

This section quantitatively compares the inference efficiency of different fusion algorithms based on the test data, and the experimental results are shown in Table 3. As can be seen from Table 4, different fusion algorithms exhibit noticeable differences in average fusion time. Compared with some lightweight deep learning-based fusion algorithms, the proposed DHAFGan requires a relatively longer average fusion time, reaching 0.5437 s. This is mainly because DHAFGan introduces a shallow–deep feature extraction module, a multi-receptive-field deep feature extraction structure, and a dual-channel hybrid attention fusion module into the network architecture. While these structures enhance the feature representation capability of the model, they inevitably increase computational complexity and inference time.

**Table 4:** Comparison of the average fusion time of the algorithm.

	ADF	FusionGAN	GANMCC	GFF	GTF	CNN	RP	HADF	DHAFGan
Dronevehicle Mean(s)	0.7892	0.1918	0.2717	1.0226	3.8966	0.2638	0.5627	**0.1463**	0.5437

Note: The bolded results are the optimal results, and the results underlined are the second-best results.

Specifically, the multi-receptive-field deep feature extraction structure can extract salient target information from infrared images and texture details from visible images at different scales, while the dual-channel hybrid attention fusion module further enhances the representation of key complementary features from both spatial and channel dimensions. Therefore, although DHAFGan has a longer average fusion time than lightweight methods such as FusionGAN, it achieves better performance in terms of information preservation, texture detail representation, and visual naturalness of the fused images. Combined with the subjective visual evaluation and objective metric results, DHAFGan demonstrates clear advantages in fusion quality. Overall, the proposed method reflects a reasonable trade-off between fusion performance and computational complexity; that is, it obtains higher-quality fusion results and stronger multimodal feature representation capability at the cost of a moderate increase in inference time.

#### External validation on the MSRS dataset.

To evaluate the generalization ability of DHAFGan, an external validation experiment was further conducted on the Multi-Spectral Road Scenarios (MSRS) dataset proposed by Tang et al. [34]. The MSRS dataset contains registered infrared-visible image pairs captured in ground-level road scenes under both daytime and nighttime conditions. The DHAFGan model trained on DroneVehicle was directly evaluated on the official MSRS test set comprising 361 image pairs, without additional training or fine-tuning. All comparison methods were evaluated on the same test images using an identical evaluation protocol. The results are presented in Table 5.

**Table 5:** Cross-dataset generalization evaluation of DHAFGan and comparison methods on the complete MSRS test set.

	SD	AG	MI	PSNR
ADF	7.8452	2.4913	1.5428	62.9716
FusionGAN	7.2164	1.7895	1.8054	58.8427
GANMcC	8.0317	1.5216	0.7284	60.3541
GFF	7.6845	2.8936	1.7927	59.6183
GTF	7.5296	2.1378	1.9642	60.9875
CNN	7.9328	3.0584	1.6159	61.8346
RP	8.0673	**3.4736**	1.5865	**62.9941**
DHAFGan	**8.2147**	3.1784	**1.9783**	62.8746

As shown in Table 5, DHAFGan exhibited a certain degree of performance degradation on the complete MSRS test set comprising 361 image pairs. Despite this decrease, DHAFGan ranked first in terms of SD and MI, second in AG, and third in PSNR among the compared methods. These results indicate that, without additional training or fine-tuning, the proposed model maintains competitive fusion performance under a different data distribution, suggesting a certain degree of cross-dataset generalization ability.

## Conclusion

This paper has addressed key limitations in existing image fusion algorithms, including inadequate perception of salient features, generated images that are inconsistent with human visual perception, and underutilization of important secondary information. To this end, we have proposed DHAFGan, a novel infrared and visible image fusion algorithm based on shallow-deep feature extraction and dual-channel hybrid attention. Firstly, The core of our framework features a hierarchical feature extraction module, which integrates a shallow feature extraction component with a deep multi-receptive field fusion unit. This design enables parallel processing of low-level detail features and cross-scale high-level semantics, achieving a more comprehensive capture of multimodal features. Secondly, a dual-channel hybrid attention fusion module (DCAFM) was designed to perform adaptive, modality-specific feature weighting for the infrared and visible light streams. By reinforcing critical information and suppressing redundant components, it ensures the precise extraction of modal features that contribute most significantly to the fusion result. Furthermore, a dual-discriminator adversarial training architecture was also constructed. This architecture facilitates the dual optimization of feature fidelity and visual consistency through adversarial training, ensuring the fused image retains source features while conforming to human visual characteristics. Finally, primary and secondary feature loss functions were formulated. These losses model the complementarity between intensity and gradient features for infrared images, while emphasizing the correlation between gradient and intensity information for visible images. This differentiated constraint strategy fully exploits the potential complementary information inherent in the dual-modal data. The experimental results show that the fused images generated by the algorithm in this paper achieve better fusion effects in subjective visual expression and are superior to seven typical fusion algorithms in objective indicators such as AG, MI and PSNR. The primary limitation of the current work is the increased computational time resulting from the model’s complexity, which affects its fusion efficiency. Therefore, our future work will focus on developing fusion algorithms that maintain high performance while significantly improving computational efficiency, potentially through network lightweighting or knowledge distillation techniques.
